# Corporal Punishment of Children in Nine Countries as a Function of Child Gender and Parent Gender

**DOI:** 10.1155/2010/672780

**Published:** 2010-09-23

**Authors:** Jennifer E. Lansford, Liane Peña Alampay, Suha Al-Hassan, Dario Bacchini, Anna Silvia Bombi, Marc H. Bornstein, Lei Chang, Kirby Deater-Deckard, Laura Di Giunta, Kenneth A. Dodge, Paul Oburu, Concetta Pastorelli, Desmond K. Runyan, Ann T. Skinner, Emma Sorbring, Sombat Tapanya, Liliana Maria Uribe Tirado, Arnaldo Zelli

**Affiliations:** ^1^Center for Child and Family Policy, Duke University, Durham, NC 27708, USA; ^2^Department of Psychology, Ateneo de Manila University, Quezon City 1108, Philippines; ^3^Queen Rania Faculty for Childhood, The Hashemite University, Zarqa 13115, Jordan; ^4^Department of Psychology, Second University of Naples, 81100 Caserta, Italy; ^5^Faculty of Psychology, Università di Roma “La Sapienza”, 00185 Rome, Italy; ^6^Child and Family Research Program in Developmental Neuroscience, Eunice Kennedy Shriver National Institute of Child Health and Human Development, Bethesda, MD 20892, USA; ^7^Department of Educational Psychology, The Chinese University of Hong Kong, Shatin, Hong Kong; ^8^Department of Psychology, Virginia Polytechnic Institute and State University, Blacksburg, VA 24061, USA; ^9^Department of Educational Psychology, Maseno University, Maseno 40105, Kenya; ^10^Department of Social Medicine, University of North Carolina, Chapel Hill, NC 27599, USA; ^11^Department of Psychology, University West, 46186 Trollhätten, Sweden; ^12^Department of Psychiatry, Chiang Mai University, Chiang Mai 50200, Thailand; ^13^Consultorio Psicológico Popular, Universidad San Buenaventura, Medellín, Colombia; ^14^Deptartment of Education Sciences, “Foro Italico”, University of Rome, 00135 Rome, Italy

## Abstract

*Background*. The purpose of this paper is to contribute to a global perspective on corporal punishment by examining differences between mothers' and fathers' use of corporal punishment with daughters and sons in nine countries. *Methods*. Interviews were conducted with 1398 mothers, 1146 fathers, and 1417 children (age range = 7 to 10 years) in China, Colombia, Italy, Jordan, Kenya, the Philippines, Sweden, Thailand, and the United States. *Results*. Across the entire sample, 54% of girls and 58% of boys had experienced mild corporal punishment, and 13% of girls and 14% of boys had experienced severe corporal punishment by their parents or someone in their household in the last month. Seventeen percent of parents believed that the use of corporal punishment was necessary to rear the target child. Overall, boys were more frequently punished corporally than were girls, and mothers used corporal punishment more frequently than did fathers. There were significant differences across countries, with reports of corporal punishment use lowest in Sweden and highest in Kenya. *Conclusion*. This work establishes that the use of corporal punishment is widespread, and efforts to prevent corporal punishment from escalating into physical abuse should be commensurately widespread.

## 1. Introduction

Prevention of physical abuse of children is a critical goal that has received attention from several leading advocates for children's rights in international contexts, including UNICEF and the World Health Organization [1]. The Convention on the Rights of the Child, which has been ratified by all except two members of the United Nations (Somalia, which has announced plans to ratify the Convention, and the United States), highlights children's right to protection from abuse and other forms of harsh treatment [2]. The Convention pays particular attention to the rights of girls because of historical and cultural precedents that condone violence against women in particular contexts.

Within this context of protecting children from abuse, parents' use of corporal punishment has increasingly come under the scrutiny of the international community. Corporal punishment can be defined as the use of physical force intended to cause pain, but not injury, for the purpose of correcting or controlling a child's behavior [3]. Attempts to reduce parents' use of corporal punishment have sometimes served as an entry point for interventions because physically abusive incidents can stem from discipline attempts that escalate into physical violence [4], and parents who use corporal punishment are at greater risk for physically abusing their children [4, 5]. For example, one recent study found that 2% of parents who did not spank their children reported child physical abuse, 6% of parents who spanked their children reported child physical abuse, and 12% of parents who hit their children with an object reported child physical abuse [5]. Although most parents who use corporal punishment do not physically abuse their children, many researchers, practitioners, and human rights organizations have called for an end to all forms of corporal punishment, in part because of the difficulty in differentiating between physical discipline and physical abuse [6]. Forms of discipline such as shaking children (especially infants) [7] and beating children with implements [8] are often classified as being physically abusive, but milder forms of discipline such as spanking or slapping also have been questioned because they can result in both physical injuries and negative psychosocial outcomes [9]. Nevertheless, bans of corporal punishment have been controversial. For many individuals, whether corporal punishment is ever justified is a moral issue. However, some researchers have argued that data regarding whether corporal punishment has negative effects on child outcomes do not warrant a ban on all forms of corporal punishment [10, 11]. 

Concern about the physical abuse of children is warranted by the prevalence and severity of the problem. In a 10-year study of emergency room visits in the United States, more than 10% of young children's blunt trauma injuries were attributed to abuse [12]. Children injured by abuse have more serious injuries, use more medical services, have longer hospital stays, and have poorer prognoses than children injured by accident do [12]. Clearly, preventing child abuse is an important public health goal.

Research has been inconsistent regarding whether parents use corporal punishment differently with daughters versus sons. Some studies report no differences in the corporal punishment of daughters versus sons, whereas other studies report that boys are more frequently corporally punished than girls [13]. Likewise, research has been inconsistent with respect to whether mothers and fathers differ in their use of corporal punishment. Some studies suggest that mothers use corporal punishment more frequently than fathers do [14], and other studies suggest more similarities than differences in mothers' and fathers' use of corporal punishment [15, 16]. 

Although many studies of corporal punishment have been conducted, nearly all have examined ethnic majority members in Western industrialized countries. By comparison, relatively little is known about patterns of corporal punishment use in distinct cultural and ethnic groups around the world. The social and legal contexts in which corporal punishment occurs vary considerably across countries. In this study, the contexts range from Sweden, in which the use of corporal punishment is illegal, to Kenya, in which the use of corporal punishment is widely accepted and used, as is the case in much of sub-Saharan Africa [17].

We compared mothers' and fathers' use of several discipline strategies that vary in severity, and we compared corporal punishment used with daughters and with sons. The samples were drawn from nine countries (China, Colombia, Italy, Kenya, Jordan, the Philippines, Sweden, Thailand, and the United States) that have been found in previous research to vary in the frequency with which parents report using physical discipline strategies [18–20]. The overarching research questions were as follows. First, across countries, what are the proportions of parents who use mild corporal punishment, use severe corporal punishment, and believe that the use of corporal punishment is necessary to rear their child? Second, do parents differ in their use of corporal punishment and in their belief about the necessity of using corporal punishment with daughters versus sons? Third, do mothers and fathers differ in the frequency with which they use corporal punishment? Fourth, is the gender composition of the parent-child dyad important, such that mother-son, mother-daughter, father-son, and father-daughter dyads differ in the frequency with which corporal punishment is used? Because we had data available from parents and children from nine countries, we also addressed the question of whether parents and children differed in their reports of the frequency with which parents used corporal punishment and whether there were differences among the countries in parents' reported use of and beliefs regarding the necessity of corporal punishment.

## 2. Materials and Methods

### 2.1. Participants

As part of the larger Parenting Across Cultures Project, 1417 families provided data. Children (age range = 7 to 10 years, M = 8.29, SD = .66; 51% girls) from all 1417 families provided data, as 1398 mothers or mother figures (age range = 19 to 70 years, M = 36.93, SD = 6.26) and 1146 fathers or father figures did (age range = 22 to 76 years, M = 39.96, SD = 6.51). Data were provided by both parents in 1127 families (80%), by just the mother in 271 families (19%), and by just the father in 19 families (1%). Eighty-two percent of the parents were married. Nonresidential parents were also asked to provide data. Ninety-seven percent of the adult respondents were the child's biological parents; the remaining 3% included stepparents, grandparents, or other adults who served as the child's main mother or father figure. In the United States, the sample was 35% European American, 33% African American, and 32% Hispanic. In Kenya, the sample was from the Luo ethnic group, which is the third largest ethnic group in Kenya (13% of the population), after the Kikuyu (22%) and Luhya (14%) ethnic groups. Although there are ethnic minorities and immigrant families to varying degrees, the samples in the other participating countries identified with the major cultural group of the country. The sample size for each country is presented in Table 1; countries did not differ by child age or gender.

Mothers, fathers, and children were recruited to participate from schools that serve socioeconomically diverse populations in each participating country: China (Jinan and Shanghai), Colombia (Medellín), Italy (Rome and Naples), Jordan (Zarqa), Kenya (Kisumu), the Philippines (Manila), Sweden (Trollhättan), Thailand (Chiang Mai), and the United States (Durham, NC). Letters describing the study were sent home with children, and parents were asked to return a signed form if they were willing to be contacted about the study (in some countries) and contacted by phone to follow up on the letter (in other countries). Rates of agreement to participate, as indicated by returning the signed form or agreeing over the telephone, ranged across sites from 24% to almost 100%. Families were then enrolled in the study until the target sample size was reached in each country. To make each country's sample as representative as possible of the city from which it was drawn, families of students from private and public schools were sampled in the approximate proportion which they represented in the population of the city. Furthermore, children were sampled from schools serving high-, middle-, and low-income families in the approximate proportion which these income groups represented in the local population. These sampling procedures resulted in an economically diverse sample that ranged from low income to high income within each site.

### 2.2. Procedures

Interviews were conducted between 2008-2009 in participants' homes, schools, or at another location chosen by the participants. Individual family members were interviewed separately so they could not hear or see one another's responses. The study measures and procedures were approved by an ethics committee in each participating country, and participants were treated ethically in accordance with the Declaration of Helsinki. Adult participants signed statements of informed consent for their own and for the target child's participation. Children signed statements of assent. In these statements, participants acknowledged that they understood that concerns about child abuse would be reported as required by law. Locally accepted practices and resources in each site were used in five cases in which interviewers became concerned that physical abuse was occurring. In addition, we had lists of available sources of help with parenting issues and other types of assistance in each site that could be conveyed to parents if the need arose.

The entire interview lasted 1.5–2 hours. Mothers and fathers were given the option of participating orally or in writing; all children were interviewed orally. Rating scales were provided in the form of visual aids to help parents and children remember the response options as they answered questions. Depending on the site, parents were given modest financial compensation for their participation, children were given small gifts, families were entered into drawings for prizes, or modest financial contributions were made to participating children's schools. The amounts varied across countries so that the compensation was appropriately motivating without being coercive.

### 2.3. Measures

#### 2.3.1. Corporal Punishment in the Last Month and Belief in Necessity of Corporal Punishment

Using items developed by UNICEF [21] for their Multiple Indicator Cluster Survey, mothers and fathers were asked whether they or anyone in their household had used each of six forms of corporal punishment with the target child in the last month. Using scoring criteria developed by UNICEF's Statistics and Monitoring Section of the Division of Policy and Practice [21], we constructed two discipline indicators. The mild physical discipline indicator reflected the proportion of parents who indicated that they or someone in their household had used one or more of the following forms of corporal punishment with the child in the last month: spanking, hitting, or slapping with a bare hand; hitting or slapping on the hand, arm, or leg; shaking; or hitting with an object. The severe physical discipline indicator reflected the proportion of parents who indicated that they or someone in their household had used one or both of the following forms of corporal punishment with the child in the last month: hitting or slapping the child on the face, head, or ears; beating the child repeatedly with an implement (this final item was not asked in the United States). An additional item was asked, “do you believe that in order to bring up (raise, educate) (target child's name) properly, you need to physically punish him/her?” Mothers and fathers responded *yes* (coded as 1) or *no* (coded as 0) for each item.

#### 2.3.2. Frequency of Corporal Punishment in the Last Year

Mothers and fathers were asked how frequently in the last year (1 = *n* *e* *v* *e* *r*, 2 = * less than once a month*, 3 = * about once a month*, 4 = *about once a week*, 5 = *almost every day*) they used two types of corporal punishment: (1) spanking, slapping, or hitting; (2) grabbing or shaking. Children were asked how frequently in the last year their mothers and their fathers disciplined them in each of those ways. Children's reports of how frequently their mothers and fathers used each form of corporal punishment were significantly correlated with mothers' and fathers' reports of how frequently they used each form of corporal punishment (range of correlations was .12 to .41; all *P* < .001).

The two sets of questions about corporal punishment used different timeframes because they were designed to elicit different kinds of information from the respondents. The dichotomous questions about whether any of the six forms of corporal punishment had been used in the last month were designed to assess recent behavior. The questions about frequency of use of two specific forms of corporal punishment within the last year were designed to capture nuances regarding frequency (because spanking less than once a month, for example, would be quite different from spanking every day in terms of its implications for the parent-child relationship and, likely, children's adjustment).

## 3. Results

Across the entire sample from all nine countries, according to at least one reporter, 54% of girls and 58% of boys had experienced mild corporal punishment, and 13% of girls and 14% of boys had experienced severe corporal punishment by their parents or someone in their household in the last month (with the caveat that parents in the United States were not asked whether they had beaten the target child with an implement). Seventeen percent of parents believed that it was necessary to use corporal punishment to rear the target child. 

Chi-square analyses were conducted to address the question of whether there were differences in parents' use of corporal punishment with girls versus boys. Table 1 shows the proportions of parents within each country who had used mild corporal punishment and severe corporal punishment with girls and boys in the last month. Because parents were reporting on their own use of corporal punishment or the use of corporal punishment by someone else in their household for the mild and severe corporal punishment items, we combined mothers' and fathers' reports so that the data in Table 1 reflect whether either parent reported use of mild or severe corporal punishment by anyone in the household. As shown, larger proportions of parents used mild corporal punishment with boys than girls in China and Kenya, and a larger proportion of parents used severe corporal punishment with boys than girls in Italy. The proportions of parents in Colombia, Jordan, the Philippines, Sweden, Thailand, and the United States who reported using corporal punishment with girls and boys in the last month did not significantly differ. 

Chi-square analyses also were conducted to address the question of whether there were differences in parents' beliefs about the necessity of using corporal punishment with boys versus girls. As shown in Table 2, larger proportions of both mothers and fathers in China believed that it was necessary to use corporal punishment with boys than with girls. Parents in the other countries did not differ significantly in their beliefs about the necessity of using corporal punishment with boys versus girls. 

The next set of analyses focused on how frequently parents had used two types of physical discipline in the last year. Repeated-measures analyses of variance were conducted separately for each country to examine differences by child and parent gender in the frequency with which parents used corporal punishment in the last year. In these analyses, parent gender was the within-subjects factor and child gender was the between-subjects factor. Interactions between parent gender and child gender were computed to examine whether there were differences in mothers' discipline of daughters versus sons compared to fathers' discipline of daughters versus sons. Table 3 shows results of these analyses based on parents' reports of the frequency with which they used corporal punishment in the last year. Table 4 shows comparable results based on children's reports of the frequency with which each of their parents had used corporal punishment on them in the last year. Overall, the means shown in Tables 3 and 4 indicate fairly infrequent use of physical punishment (i.e., less than once a month to about once a month).

As shown in Table 3, in seven of the nine countries, mothers reported spanking, slapping, or hitting their target child significantly more frequently than fathers did in the same families. In Colombia, this parent-gender effect was qualified by a significant interaction with child gender, *F*(1, 106) = 7.40, *P* < .01, such that mothers reported spanking, slapping, or hitting daughters more frequently than sons, whereas fathers reported spanking, slapping, or hitting sons more frequently than daughters. In Kenya, this parent-gender effect was qualified by a significant interaction with child gender, *F*(1, 98) = 4.31, *P* < .05, such that mothers reported spanking, slapping, or hitting sons and daughters with equal frequency, but fathers reported spanking, slapping, or hitting sons more frequently than daughters. Only in Sweden (where any spanking, slapping, or hitting at all was reported by only 5 parents) and in Thailand were there no significant differences in the frequency with which mothers and fathers reported spanking, slapping, or hitting their target child. There was a main effect of child gender in China and Jordan; in both countries, sons were spanked, slapped, or hit more frequently than daughters. With respect to grabbing or shaking the child, mothers reported more frequently using this discipline strategy than fathers did in Colombia and Italy, whereas fathers reported more frequently using this discipline strategy than mothers did in Sweden. There was a main effect of child gender on frequency of grabbing or shaking in Jordan and the United States; in both countries, boys were grabbed and shaken more frequently than girls.

As shown in Table 4, there was a significant main effect of parent gender on spanking, slapping, or hitting in Italy, Jordan, and Kenya. Kenyan and Jordanian children reported that their mothers spanked, slapped, or hit them more frequently than their did fathers. In Italy, the main effect of parent gender was qualified by a significant Parent Gender X Child Gender interaction; boys in Italy reported that their mothers and fathers spanked, slapped, or hit them with equal frequency, whereas girls in Italy reported that their mothers spanked, slapped, or hit them more frequently than their fathers did, *F*(1, 196) = 6.00, *P* < .05. There was no significant main effect of parent gender in Colombia or the Philippines, but there were significant Parent Gender X Child Gender interactions in both countries; boys in Colombia and the Philippines reported that their fathers spanked, slapped, or hit them more frequently than their mothers did, whereas girls in Colombia and the Philippines reported that their mothers spanked, slapped, or hit them more frequently than their fathers did, *F*(1, 106) = 5.34 in Colombia and *F*(1,104) = 6.13 in the Philippines, both *P* *'* *s* < .05. With respect to grabbing or shaking, children in China and Sweden reported that their fathers grabbed or shook them more than their mothers did, whereas children in Kenya reported that their mothers grabbed or shook them more than their fathers did. In Sweden and the United States, boys reported that their parents had grabbed or shaken them more frequently in the last year than girls reported. 

We next compared children's reports of how frequently their mothers and fathers corporally punished them with mothers' and fathers' reports of how frequently they corporally punished their children. Results of paired samples *t*-tests are shown in Table 5. In only two of the countries (China and Kenya) were there significant differences between children's and their mothers' reports of the frequency with which mothers spanked, slapped, or hit the child; there were no differences between children's and their fathers' reports of the frequency with which fathers spanked, slapped, or hit the child. There were more differences between children's and parents' reports related to how frequently the parents grabbed or shook the child, with significant differences between children's and mothers' reports in six of the nine countries (China, Colombia, Italy, Kenya, Thailand, and the United States) and significant differences between children's and fathers' reports in three countries (China, Colombia, and Kenya). Across the two forms of corporal punishment and all nine countries, eight of the differences reflected parents reporting using corporal punishment more frequently than their children reported that they did, whereas three of the differences reflected children reporting that their parents used corporal punishment more frequently than their parents reported that they did. However, for 25 of the 36 tests, there were no significant differences between children's and their parents' reports of how frequently the parents used the two forms of corporal punishment.

Finally, we conducted multivariate analyses of variance to test for differences across countries in the mean levels of each item reflecting reported use of and beliefs about the necessity of using corporal punishment. As shown in Table 6, there were significant country differences on all 14 variables of interest. On most variables and for mothers', fathers', and children's reports, reported use of and belief in the necessity of using corporal punishment were the lowest in Sweden and the highest in Kenya. The exception was for grabbing and shaking, which were most frequent in Jordan, according to mothers', fathers', and children's reports. Between these two anchor points, the other countries differed from one another on many of the variables, with the specific patterns of significant differences depending on the construct and the reporter.

## 4. Discussion

In samples of mothers, fathers, and children from nine countries, we found that, according to at least one reporter, 54% of girls and 58% of boys had experienced mild corporal punishment, and 13% of girls and 14% of boys had experienced severe corporal punishment by their parents or someone in their household in the last month. Seventeen percent of parents believed that it was necessary to use corporal punishment to rear the target child. Although levels of prevalence in many of the countries were relatively high, corporal punishment was generally used infrequently (about once a month or less frequently). Mothers generally used corporal punishment more frequently than fathers did. When child gender differences were found, parents and children reported that parents in the same family used corporal punishment more frequently with boys than with girls; only in China was there a difference in parents' beliefs about the necessity of using corporal punishment with girls versus boys, with a larger proportion of parents believing that it was necessary to use corporal punishment with boys than with girls. Although there were statistical main effects of parent gender and child gender, there were few significant statistical interactions between parent gender and child gender in the prediction of the use of corporal punishment. In two exceptions, there were significant interactions between child and parent genders. Kenyan mothers reported using corporal punishment equally frequently with daughters and sons, whereas Kenyan fathers reported using corporal punishment less frequently with daughters than with sons, which is consistent with some cultural constraints in Kenya regarding fathers' use of corporal punishment with daughters [22]. In addition, Colombian mothers were found to report using corporal punishment less frequently with sons than with daughters, whereas Colombian fathers were found to report using corporal punishment less frequently with daughters than with sons. This significant interaction between child and parent genders also emerged when children reported on their parents' use of corporal punishment. In particular, boys in Colombia and the Philippines reported that their fathers spanked, slapped, or hit them more frequently than their mothers did, whereas girls in Colombia and the Philippines reported that their mothers spanked, slapped, or hit them more frequently than their fathers did. In the cultural context of Colombia and the Philippines where gender roles are clearly demarcated [23, 24], the behavior of the same-sex parent may have been more salient, attended to, and easily remembered by children.

Previous research has shown variation across studies with respect to whether girls and boys differ in the frequency with which they are corporally punished, but when differences are found, boys are generally punished more frequently than girls [13]. Results from this study suggest that perhaps one reason for the variation in prior research is that whether there are gender differences in the frequency of corporal punishment depends on the cultural context in which the family lives. A question for future research will be whether boys and girls are affected by corporal punishment in the same way. With respect to parent gender, one reason that mothers and fathers may differ in the frequency with which they use corporal punishment is that, on average, mothers spend more time with children and have more responsibility for their day-to-day care than fathers do [25], so mothers may more often be in a position to witness misbehavior and have more opportunity to respond. 

In all nine countries, the proportion of parents who reported that it is necessary to use corporal punishment to rear the target child was smaller than the proportion of parents who said that they had used corporal punishment in the last month. This disconnect between parents' beliefs and behaviors in disciplining their children suggests a possible entry point for working with parents to reduce their use of corporal punishment, starting with their belief that it is not necessary to use corporal punishment. 

There also were differences across countries in parents' use of corporal punishment. Kenya, Jordan, the Philippines, Italy, and Colombia showed the highest incidence and frequency of mild corporal punishment according to mother, father, and child reports. To understand what sets these countries apart from the countries with lower incidence and frequency of mild corporal punishment, one can consider what characteristics are common among these countries in terms of cultural values, child-rearing practices, and so forth. For example, future research could investigate whether the countries that are the highest in parents' use of corporal punishment are also the highest in their emphasis on parental authority and child obedience. Our results also suggest that broad cultural generalizations (e.g., individualist versus collectivist frameworks) are insufficient for understanding parenting in these underresearched cultural contexts. 

Research on effects of corporal punishment on children's adjustment remains controversial. Although some scholars conclude that the bulk of the evidence suggests that corporal punishment predicts worse outcomes for children [9], other scholars conclude that corporal punishment is not necessarily predictive of worse outcomes for children if it is administered within certain parameters (e.g., not too harsh, not too frequent) [10, 11]. The normativeness of corporal punishment both historically [26] and culturally [20, 27] may also affect how corporal punishment is related to children's adjustment.

This study focused on corporal punishment during middle childhood (ages 7–10 years). Children's age is important in understanding the implications of particular forms of corporal punishment. For example, infants and toddlers were not included in this sample; therefore, “shook” was classified as “mild” rather than “severe.” If an infant is shaken, that form of corporal punishment can be severe, even lethal. Previous research also suggests that, although there is controversy regarding whether spanking children ages 2–6 years leads to positive as well as negative outcomes [11, 27], spanking adolescents is more consistently found to relate to negative outcomes. Because our sample ranged in age from 7 to 10 years, caution should be used in attempting to generalize the findings to older or younger children.

This study has several limitations. The findings reported should be interpreted as subjective perceptions of the frequency of corporal punishment, which may have been biased by either over- or underreporting. In our tests of differences between parents' and children's reports of the frequency with which parents used two forms of corporal punishment, there were no significant differences for 25 of the 36 tests conducted; in the remaining tests, parents sometimes reported that they used corporal punishment more frequently than their children reported that they did, and children sometimes reported that their parents used corporal punishment more frequently than their parents reported that they did. Neither parents nor children are necessarily more accurate reporters than the others; rather, theirs are two different perspectives on one aspect of parent-child interactions. Parents, in particular, may have under-reported their use of corporal punishment if they were aware of social proscriptions against its use, but in only 3 of 36 tests did children report that their parents used corporal punishment more frequently than their parents reported. Another limitation is that the samples in this study cannot be considered nationally representative of the countries from which they were drawn; therefore, caution should be used in attempting to generalize the findings. This caution is especially important in relation to the interpretation of the cross-national comparisons of the reported use of corporal punishment and parents' beliefs about the necessity of using corporal punishment to rear the target child.

Sweden was the first country to pass legislation outlawing the use of corporal punishment. The legislation aimed to change attitudes regarding the use of physical force against children as a first step to reduce the use of corporal punishment of children, to offer parents and professionals a clear set of guidelines and to lead to earlier identification and intervention in cases of abuse [28]. In this study, only a small proportion of Swedish parents and children reported any form of corporal punishment, and no Swedish parents reported believing that using corporal punishment was necessary to rear the target child; it is possible that these were underreports given that parents may have been reluctant to report engaging in an illegal behavior (although there were no significant differences between Swedish children's and parents' reports of the frequency with which parents used corporal punishment). In the cross-country comparisons, Sweden was consistently significantly lower than the other countries in mothers', fathers', and children's reports of parents' use of corporal punishment. Another approach to reducing parents' use of corporal punishment is to change community norms related to the use of corporal punishment, even in the absence of legislation banning its use [29]. Italy has moved in this direction; in particular, in 1996, the Italian Supreme Court ruled that corporal punishment was unlawful although there is no legislation outlawing the use of corporal punishment in Italy. Although the use of corporal punishment in Italy was relatively high, Italian parents were second to Swedish parents in their belief in the necessity of using corporal punishment to rear the target child. In several countries, changes in norms have preceded legislation banning corporal punishment [28]. 

In part, as a response to the Convention on the Rights of the Child, several countries have implemented parenting programs that attempt to reduce parents' use of corporal punishment. One approach to reducing parents' use of corporal punishment has been to implement preventive interventions that aim to reduce parental stress, substance use, and poverty and to increase parents' access to supportive services [30]. For example, preventive interventions such as nurse home visiting programs with primiparous mothers reduce child abuse [31]. In the Philippines, Parent Effectiveness Service is a multifaceted parenting program that includes information designed to help parents manage the behavior of their young children [32]. Likewise, reducing parents' use of corporal punishment was one goal of a parenting program implemented in Thailand by the Ministry of Education [32]. Corporal punishment appears to be used by many parents in both the Philippines and Thailand although its use is infrequent. It remains to be seen whether fewer parents will use corporal punishment over time. One of the most widely publicized aspects of the social and legal context in China is the one-child policy, which, in conjunction with the social value placed on sons, may have contributed to the greater use of corporal punishment with sons than with daughters (e.g., because there is greater pressure for single sons to behave well and attain high levels of achievement). 

In the United States, the American Academy of Pediatrics issued a policy statement that the use of corporal punishment is of “limited effectiveness and has potentially deleterious side effects” and recommended that “parents be encouraged and assisted in the development of methods other than spanking for managing undesired behavior” [33, page 723]. In addition, the American Academy of Pediatrics recommends anticipatory guidance as a way for pediatric primary care physicians to communicate with parents about age-appropriate topics that can optimize parents' care for their children [34]. By discussing with parents children's misbehaviors and parents' disciplinary responses, pediatricians have the opportunity to strategize with parents about how to respond best to children's misbehaviors.

## 5. Conclusions

There was considerable variability in proportions of mothers, fathers, and children in China, Colombia, Italy, Kenya, Jordan, the Philippines, Sweden, Thailand, and the United States who reported the use of corporal punishment and believed that the use of corporal punishment is necessary to rear the target child. Overall, mothers reportedly used corporal punishment more frequently than fathers, and sons were reportedly more frequently corporally punished than daughters were. International efforts to eliminate child abuse and promote children's right to protection will be both challenging and important because of the prevalence of corporal punishment.

## Figures and Tables

**Table 1:** Percentages of parents reporting two types of corporal punishment in the last month.

	Mild corporal punishment			Severe corporal punishment		
	Girls	Boys	*χ* ^2^	Girls	Boys	*χ* ^2^
China (*n* = 241)	48	60	3.25*	10	15	1.10
Colombia (*n* = 108)	68	63	.40	15	4	3.42
Italy (*n* = 203)	61	66	.41	12	23	4.31*
Jordan (*n* = 114)	66	80	2.99	21	31	1.58
Kenya (*n* = 100)	82	97	5.39*	61	62	.01
Philippines (*n* = 120)	71	77	.54	9	8	.00
Sweden (*n* = 101)	9	6	.34	0	0	N/A
Thailand (*n* = 120)	58	72	2.77	5	3	.25
U.S.A. (*n* = 310)	38	36	.13	4	5	.30
Full sample (*N* = 1417)	54	58	2.60	13	14	.36

*P < .05.

*Note*. Values reflect the percentages of families in which either the mother or father reports that either the mother or father or anyone in the household has used mild corporal punishment and severe corporal punishment in the last month and chi-square tests of differences by child gender. Mild corporal punishment included spanking, hitting, or slapping with a bare hand; hitting or slapping on the hand, arm, or leg; shaking; or hitting with an object. Severe corporal punishment included: hitting or slapping the child on the face, head, or ears; beating the child repeatedly with an implement (beating was not asked in the U.S.A.).

**Table 2:** Percentages of parents reporting belief that corporal punishment is necessary to rear the target child.

	Mothers' belief			Fathers' belief		
	Girls	Boys	*χ* ^2^	Girls	Boys	*χ* ^2^
China	14	36	14.91*	20	33	4.97*
Colombia	14	19	.53	13	8	.68
Italy	5	4	.09	2	4	.27
Jordan	8	7	.05	8	10	.16
Kenya	44	56	1.41	48	54	.38
Philippines	13	20	1.19	16	15	.03
Sweden	0	0	N/A	0	0	N/A
Thailand	16	11	.63	22	10	2.39
U.S.A.	17	13	.87	11	16	.70
Full sample	15	17	1.90	16	17	.29

*P < .05.

*Note*. Values reflect the percentages of parents who reported that they believe that using corporal punishment is necessary to rear the target child and Chi-square tests of differences by child gender.

**Table 3:** Means (standard deviations) for parents' reports of the frequency of their corporal punishment use in last year.

	Spank, slap, or hit						Grab or shake					
	Mother	Father	Parent gender *F *	Daughter	Son	Child gender *F*	Mother	Father	Parent gender *F*	Daughter	Son	Child gender *F*
China	1.85 (.92)	1.67 (.80)	7.37*	1.65 (.65)	1.88 (.68)	7.59*	1.42 (.74)	1.45 (.75)	.16	1.40 (.51)	1.47 (.56)	.76
Colombia	2.08 (1.15)	1.75 (.98)	6.47*	1.99 (.89)	1.82 (.82)	1.02	1.92 (1.12)	1.56 (.87)	7.69*	1.65 (.68)	1.84 (.84)	1.75
Italy	2.22 (1.16)	1.80 (.95)	28.11*	1.93 (.93)	2.11 (.90)	1.39	2.09 (1.21)	1.78 (1.01)	5.87*	1.97 (.95)	1.99 (.94)	.29
Jordan	2.54 (1.20)	2.20 (1.09)	10.38*	2.10 (.99)	2.61 (.98)	8.53*	2.42 (1.46)	2.31 (1.39)	.74	2.01 (1.17)	2.61 (1.33)	5.32*
Kenya	3.36 (.86)	2.85 (.97)	14.30*	3.01 (.73)	3.26 (.61)	3.11	1.84 (1.12)	1.85 (1.22)	.12	1.78 (.86)	1.95 (.89)	.90
Philippines	2.36 (1.19)	2.13 (1.15)	4.06*	2.19 (1.08)	2.34 (.98)	.10	1.79 (1.06)	1.64 (1.08)	.49	1.74 (.92)	1.72 (.78)	.01
Sweden	1.03 (.17)	1.03 (.16)	.37	1.05 (.19)	1.02 (.14)	3.65	1.39 (.65)	1.51 (.74)	6.02*	1.39 (.53)	1.53 (.75)	.17
Thailand	1.80 (.92)	1.65 (.95)	1.59	1.67 (.75)	1.84 (.87)	1.13	1.24 (.52)	1.26 (.69)	.02	1.25 (.44)	1.24 (.54)	.31
U.S.A.	1.86 (.96)	1.61 (.81)	4.13*	1.79 (.86)	1.81 (.89)	.24	1.31 (.70)	1.27 (.67)	.14	1.23 (.55)	1.37 (.69)	5.95*

*P < .05.

*Note*. Values are means, standard deviations, and *F*-tests from repeated measures analyses of variance with parent gender as the within-subjects factor and child gender as the between-subjects factor. Significant Parent Gender X Child Gender interactions for spanking, slapping, or hitting in Colombia and Kenya are described in the text. Frequency of corporal punishment was rated as 1 = never, 2 = less than once a month, 3 = about once a month, 4 = about once a week, and 5 = almost every day.

**Table 4:** Means (standard deviations) for children's reports of the frequency of their parents' corporal punishment use in the last year.

	Spank, slap, or hit						Grab or shake					
	Mother	Father	Parent gender *F*	Daughter	Son	Child gender *F*	Mother	Father	Parent gender *F*	Daughter	Son	Child gender *F*
China	1.56 (.94)	1.62 (.93)	.75	1.51 (.73)	1.68 (.86)	2.64	1.18 (.62)	1.26 (.64)	6.39*	1.16 (.48)	1.27 (.56)	2.58
Colombia	1.92 (1.20)	1.72 (1.11)	2.84	1.83 (.98)	1.80 (1.10)	.02	1.26 (.75)	1.21 (.61)	.32	1.28 (.65)	1.18 (.54)	.82
Italy	2.13 (1.38)	1.96 (1.23)	4.35*	2.03 (1.19)	2.06 (1.16)	.35	1.55 (1.12)	1.58 (1.13)	.18	1.48 (.91)	1.65 (1.16)	1.27
Jordan	2.64 (1.23)	2.21 (1.13)	16.20*	2.38 (1.08)	2.48 (.99)	.32	2.18 (1.41)	2.08 (1.24)	1.04	1.92 (1.20)	2.34 (1.20)	2.91
Kenya	3.64 (.91)	3.06 (1.05)	23.78*	3.29 (.79)	3.45 (.86)	.93	1.50 (.97)	1.33 (.67)	4.14*	1.32 (.58)	1.56 (.84)	2.93
Philippines	2.26 (1.32)	2.23 (1.36)	.08	2.15 (1.26)	2.39 (1.24)	1.28	1.76 (1.22)	1.65 (1.14)	3.76	1.65 (1.18)	1.75 (1.06)	.92
Sweden	1.10 (.53)	1.14 (.57)	3.49	1.15 (.54)	1.06 (.29)	1.18	1.49 (.97)	1.61 (1.11)	5.86*	1.29 (.68)	1.77 (1.15)	5.27*
Thailand	1.84 (1.20)	1.90 (1.28)	.22	1.86 (1.22)	1.87 (1.06)	.00	1.46 (1.02)	1.36 (.93)	1.50	1.39 (.85)	1.43 (.89)	.05
U.S.A.	1.89 (1.21)	1.71 (1.09)	3.68	1.81 (1.11)	1.86 (1.09)	.53	1.47 (1.01)	1.41 (.91)	.10	1.40 (.87)	1.52 (.97)	4.81*

*P < .05.

*Note*. Values are means, standard deviations, and *F*-tests from repeated measures analyses of variance with parent gender as the within-subjects factor and child gender as the between-subjects factor. Significant Parent Gender X Child Gender interactions for spanking, slapping, or hitting in Colombia, Italy, and the Philippines are described in the text. Frequency of corporal punishment was rated as 1 = never, 2 = less than once a month, 3 = about once a month, 4 = about once a week, and 5 = almost every day.

**Table 5:** Means (standard deviations) for comparisons of parents' and children's reports of the frequency of parents' corporal punishment use in last year.

	Spank, slap, or hit						Grab or shake					
	Mother	Child	*t*	Father	Child	*t*	Mother	Child	*t*	Father	Child	*t*
China	1.85 (.92)	1.56 (.94)	3.69*	1.66 (.80)	1.62 (.93)	.69	1.42 (.74)	1.16 (.57)	4.37*	1.45 (.75)	1.26 (.64)	3.00*
Colombia	2.08 (1.15)	1.92 (1.20)	1.11	1.75 (.98)	1.72 (1.11)	.20	1.92 (1.12)	1.26 (.75)	5.04*	1.56 (.87)	1.21 (.61)	3.68*
Italy	2.22 (1.17)	2.13 (1.38)	.77	1.80 (.95)	1.99 (1.26)	−1.93	2.09 (1.21)	1.55 (1.12)	4.93*	1.79 (1.01)	1.62 (1.15)	1.72
Jordan	2.55 (1.21)	2.65 (1.22)	−.79	2.19 (1.09)	2.21 (1.12)	−.15	2.40 (1.47)	2.22 (1.41)	1.13	2.30 (1.38)	2.07 (1.24)	1.51
Kenya	3.36 (.86)	3.64 (.91)	−2.48*	2.85 (.97)	3.06 (1.05)	−1.75	1.84 (1.12)	1.50 (.97)	2.43*	1.85 (1.22)	1.33 (.67)	3.69*
Philippines	2.36 (1.19)	2.25 (1.31)	.77	2.13 (1.15)	2.30 (1.31)	−.99	1.79 (1.06)	1.75 (1.22)	.25	1.64 (1.08)	1.65 (1.12)	−.07
Sweden	1.03 (.17)	1.06 (.37)	−.73	1.03 (.16)	1.17 (.65)	−1.95	1.39 (.65)	1.46 (.94)	−.66	1.51 (.74)	1.64 (1.17)	−.95
Thailand	1.80 (.92)	1.88 (1.22)	−.59	1.65 (.95)	1.82 (1.21)	−1.18	1.24 (.52)	1.48 (1.04)	−2.12*	1.26 (.69)	1.37 (.92)	−.84
U.S.A.	1.86 (.96)	1.90 (1.22)	−.51	1.59 (.79)	1.65 (1.01)	−.69	1.31 (.70)	1.47 (1.01)	−2.49*	1.27 (.67)	1.30 (.75)	−.34

*P < .05.

*Note*. Values are means, standard deviations, and paired samples *t*-tests. Frequency of corporal punishment was rated as 1 = never, 2 = less than once a month, 3 = about once a month, 4 = about once a week, and 5 = almost every day.

**Table 6:** Means (standard deviations) for country comparisons of corporal punishment use and beliefs.

	China	Colombia	Italy	Jordan	Kenya	Philippines	Sweden	Thailand	U.S.A.	*F*
Mother reports										
Proportion used mild cp in last month	.16^a^ (.25)	.26^bc^ (.31)	.26^be^ (.28)	.33^bg^ (.33)	.43^bdfi^ (.32)	.31^bk^ (.31)	.01^bdfjhlm^ (.06)	.21^hjno^ (.25)	.12^dfhjlnp^ (.20)	28.48*
Proportion used severe cp in last month	.04^a^ (.15)	.03^a^ (.14)	.07^c^ (.17)	.12^b^ (.25)	.31^bd^ (.37)	.04^a^ (.13)	.00^a^ (.00)	.02^a^ (.10)	.04^a^ (.19)	26.14*
Proportion believe necessary to use cp	.25^ac^ (.43)	.16^aj^ (.37)	.05^ade^ (.21)	.07^ad^ (.26)	.49^b^ (.50)	.16^ag^ (.37)	.00^adhi^ (.00)	.13^a^ (.34)	.15^adfj^ (.36)	19.61*
Frequency spank, slap, hit in last year	1.85^ace^ (.92)	2.07^acg^ (1.15)	2.22^acfi^ (1.17)	2.54^acfhk^ (1.21)	3.36^bc^ (.86)	2.36^acfi^ (1.20)	1.03^ad^ (.17)	1.80^acej^ (.92)	1.86^acjl^ (.96)	43.26*
Frequency grab or shake in last year	1.41^a^ (.74)	1.90^bc^ (1.11)	2.09^be^ (1.21)	2.39^bdg^ (1.46)	1.84^bc^ (1.12)	1.79^bhi^ (1.06)	1.39^dfh^ (.65)	1.24^dfhj^ (.52)	1.30^dfhj^ (.70)	25.69*

Father reports										
Proportion used mild cp in last month	.13^ace^ (.21)	.21^acg^ (.30)	.18^aci^ (.26)	.28^acfgi^ (.31)	.47^bc^ (.35)	.27^acfk^ (.28)	.01^ad^ (.05)	.18^ac^ (.25)	.09^ahjl^ (.18)	26.52*
Proportion used severe cp in last month	.04^a^ (.14)	.03^a^ (.12)	.03^a^ (.12)	.07^ac^ (.20)	.25^b^ (.34)	.01^a^ (.05)	.00^a^ (.00)	.00^ad^ (.00)	.01^a^ (.12)	25.16*
Proportion believe necessary to use cp	.26^ac^ (.44)	.11^ad^ (.32)	.03^ad^ (.17)	.09^ad^ (.29)	.50^b^ (.50)	.16^a^ (.37)	.00^ad^ (.00)	.16^a^ (.37)	.13^ad^ (.34)	20.25*
Frequency spank, slap, hit in last year	1.66^ace^ (.80)	1.75^acg^ (.98)	1.78^acg^ (.94)	2.20^acfh^ (1.09)	2.85^bc^ (.97)	2.15^acfi^ (1.16)	1.03^ad^ (.16)	1.65^acgj^ (.95)	1.60^acgj^ (.81)	29.21*
Frequency grab or shake in last year	1.45^ac^ (.75)	1.56^a^ (.87)	1.79^ad^ (1.01)	2.33^b^ (1.38)	1.85^ad^ (1.22)	1.65^a^ (1.09)	1.51^a^ (.75)	1.26^ac^ (.69)	1.28^ac^ (.68)	14.32*

Child reports										
Frequency mother spank, slap, hit in last year	1.56^ace^ (.94)	1.92^acg^ (1.20)	2.14^acfg^ (1.37)	2.61^acfh^ (1.22)	3.64^bc^ (.91)	2.31^acfi^ (1.31)	1.10^ad^ (.54)	1.86^acg^ (1.21)	1.83^acgj^ (1.15)	45.08*
Frequency mother grab or shake in last year	1.18^ac^ (.62)	1.26^ae^ (.75)	1.56^ad^ (1.12)	2.18^b^ (1.41)	1.50^a^ (.97)	1.79^df^ (1.23)	1.45^a^ (.95)	1.47^a^ (1.03)	1.44^a^ (.96)	11.79*
Frequency father spank, slap, hit in last year	1.62^ace^ (.93)	1.72^acg^ (1.11)	1.96^acf^ (1.23)	2.20^acfi^ (1.13)	3.06^bc^ (1.05)	2.30^acfhi^ (1.35)	1.14^ad^ (.57)	1.90^ac^ (1.28)	1.72^acj^ (1.09)	25.86*
Frequency father grab or shake in last year	1.26^ac^ (.64)	1.21^ac^ (.61)	1.58^ad^ (1.13)	2.08^b^ (1.24)	1.33^a^ (.67)	1.63^ad^ (1.12)	1.61^a^ (1.11)	1.36^a^ (.93)	1.43^a^ (.92)	9.84*

*P < .05.

*Note*. Values are means, standard deviations, and *F* statistics from multivariate analyses of variance. Frequency of corporal punishment was rated as 1 = never, 2 = less than once a month, 3 = about once a month, 4 = about once a week, and 5 = almost every day. Post-hoc pairwise comparisons with Bonferroni corrections for multiple comparisons showed that means designated with ^a^ differ significantly from ^b^, ^c^ differ from ^d^, ^e^ differ from ^f^, ^g^ differ from ^h^, ^i^ differ from ^j^, ^k^ differ from ^l^, ^m^ differ from ^n^, and ^o^ differ from ^p^.
